# Participant experiences of guided self-help Acceptance and Commitment Therapy for improving quality of life in muscle disease: a nested qualitative study within the ACTMus randomized controlled trial

**DOI:** 10.3389/fpsyg.2023.1233526

**Published:** 2023-12-01

**Authors:** Victoria Edwards, Chiara Vari, Michael Rose, Christopher D. Graham, Nicola O'Connell, Emma Taylor, Lance M. McCracken, Aleksandar Radunovic, Wojtek Rakowicz, Sam Norton, Trudie Chalder

**Affiliations:** ^1^Department of Psychological Medicine, Institute of Psychiatry, Psychology and Neuroscience, King's College London, London, United Kingdom; ^2^Department of Neurology, King's College Hospital, London, United Kingdom; ^3^School of Psychological Sciences and Health, University of Strathclyde, Glasgow, United Kingdom; ^4^Department of Psychology, Uppsala University, Uppsala, Sweden; ^5^Barts and the London MND Centre, Royal London Hospital, London, United Kingdom; ^6^Wessex Neurological Service, University Hospital Southampton, Southampton, United Kingdom; ^7^Department of Psychology, Institute of Psychiatry, Psychology and Neuroscience, King's College London, London, United Kingdom; ^8^Department of Inflammation Biology, Centre for Rheumatic Disease, Faculty of Life Sciences and Medicine, King's College London, Weston Education Centre, London, United Kingdom

**Keywords:** Acceptance and Commitment Therapy, muscle disorders, facioscapulohumeral muscular dystrophy, limb-girdle dystrophy, inclusion body myositis, talking therapies, quality of life, mood

## Abstract

**Introduction:**

In adults, muscle disease (MD) is typically a chronic long-term condition that can lead to a reduced quality of life (QoL). Previous research suggests that a psychological intervention, in particular Acceptance and Commitment Therapy (ACT), may help improve QoL for individuals living with chronic conditions such as MD.

**Methods:**

This nested qualitative study was incorporated within a randomized controlled trial which evaluated a guided self-help ACT intervention for people living with MD to explore their experiences of the intervention. Semi-structured interviews (*n* = 20) were conducted with those who had received ACT. Data were analyzed via thematic analysis.

**Results:**

There were four overarching themes. (1) Views on whether therapy sessions would help with a medical condition: participants' expectations regarding ACT varied. Some participants were skeptical about mindfulness. (2) I was able to look at things in a different way: participants described increased meaningful activity, greater awareness of thoughts and emotions and acceptance or adaptation to mobility problems. Some described improvement in the quality of relationships and a sense of feeling free. (3) Treating the body and the mind together: following the intervention participants noted that a holistic approach to healthcare is beneficial. (4) Intervention delivery: The remote delivery was generally seen as helpful for practical reasons and allowed participants to speak openly. Participants voiced a need for follow-up sessions.

**Discussion:**

Overall, the intervention was experienced as acceptable. Suggested improvements included de-emphasizing the role of mindfulness and adding follow-up sessions.

## Introduction

Muscle diseases (MDs), such as limb-girdle muscular dystrophy, facioscapulohumeral dystrophy, and inclusion body myositis, are primary disorders of the muscle that can be acquired or genetic. In the UK, MDs have an estimated prevalence of 29.5 per 100,000—equating to 110,000 children and adults (Carey et al., 2021). Across 19 countries, the combined prevalence for all muscular dystrophies ranges from 19.8 and 25.1 per 100,000 person-years (Theadom et al., 2015). They are usually chronic and progressive and are currently without a definitive cure. They involve muscle wasting and weakness, which causes insidious declines in mobility and physical functioning. Many with MD eventually require mobility aides, including wheelchairs and orthoses. MDs are also associated with chronic pain, sleep disturbance, and fatigue (Merrison and Hanna, 2009; Della Marca et al., 2013). Given these challenges, it is unsurprising that on average people with MD experience reduced quality of life (QoL) (Graham et al., 2011), and some experience anxiety and depression (Kelly et al., 2022).

However, the QoL of individuals with MD is not only affected by disease severity or physical disability (Graham et al., 2011, 2014; Burns et al., 2012; Rose et al., 2012). A wide range of psychological variables, including illness beliefs (Graham et al., 2013, 2014), coping methods (Ahlstrom and Sjoden, 1996; Natterlund et al., 2001; Graham et al., 2014), and psychological flexibility (Graham C. D. et al., 2016), significantly contribute to QoL. This presents the possibility that interventions designed to improve key psychological factors could improve QoL in MD, even in the absence of disease-modifying treatments (Graham et al., 2015). Given the limited research into psychological interventions for MD, we set out to develop and trial a psychological intervention for improving QoL in MD (Thompson et al., 2022). Among candidate approaches, we chose Acceptance and Commitment Therapy (ACT). ACT is designed to improve a psychological process called “psychological flexibility,” which, in a previous observational study in MD, was predictive of QoL (Graham C. D. et al., 2016). Psychological flexibility can be defined as “[…] the capacity to persist or to change behavior in a way that includes conscious and open contact with thoughts and feelings, appreciates what the situation affords, and serves one's goals and values” (McCracken and Vowles, 2014). Many have argued that ACT is particularly suited to helping people live well with chronic diseases such as MD (Graham et al., 2015), motor neurone disease (Gould et al., 2015), and multiple sclerosis (Thompson et al., 2022). A pragmatic benefit of ACT is that it aims to help participants live well in contexts where uncomfortable thoughts and emotions reflect an objectively difficult situation, such as living with a chronic disease (Graham C. et al., 2016). ACT differs from CBT in that it is concerned with people's relationship with thoughts rather than the content of thoughts. ACT also includes acceptance-based emotion regulation strategies, which are encouraged when acceptance of difficult feelings facilitates life-enriching activity (e.g., showing willingness to experience the anxiety that accompanies using a wheelchair, to facilitate traveling to meet friends for coffee).

Following a promising preliminary pilot case series of a guided self-help intervention with people with MD (Graham et al., 2017), we undertook the “ACTMus” randomized controlled trial (Rose et al., 2018) to evaluate the efficacy of the intervention for improving QoL. We recruited 155 participants with one of four MDs—limb-girdle muscular dystrophy (LGMD), Becker's muscular dystrophy (BMD; dystrophin deficiency), facioscapulohumeral muscular dystrophy (FSHD), and inclusion body myositis (IBM). Participants were randomized to receive either standard medical care plus the guided self-help ACT programme, or standard medical care only. The results showed that ACT plus standard medical care was superior to standard medical care alone, with moderate to large effect sizes for QoL and for secondary outcomes such as mood and functional impairment (Rose et al., 2022).

While the aforementioned quantitative data are important for understanding whether the guided self-help ACT intervention is efficacious, additional useful information can be gained by examining the experience of those who participated in the intervention. A qualitative study nested within a clinical trial can offer a greater understanding of the acceptability of the intervention and other treatment effects, intended or otherwise, not captured by standardized quantitative measures. It can also provide clues regarding barriers and facilitators to engagement and key treatment processes (Lewin et al., 2009; O'Cathain et al., 2014).

There have been several nested qualitative studies of remote guided self-help ACT interventions in long-term health conditions—including people living with chronic pain and carers of people living with cancer and dementia (Köhle et al., 2017; Bendelin et al., 2020; Contreras et al., 2022). In all, participants described making conscious changes to their behavior in line with the ACT model. Experiences of the remote guided self-help configurations varied. While some appreciated the flexibility this configuration offers (Köhle et al., 2017; Bendelin et al., 2020), others felt it introduced the pressure of having to work to a deadline (Bendelin et al., 2020). There was a range of responses to the lower-intensity brief input from therapists. Some participants felt understood and supported, whereas others had hoped for deeper, more personalized discussions.

In the context of the ACTMus trial, the present nested qualitative study was designed to provide insight into the experiences of those receiving this intervention. We were particularly interested in participant descriptions of the outcomes of treatment, barriers and facilitators to engagement, and experiences of the self-help and remote configuration.

## Materials and methods

### Design

A nested qualitative study was conducted as part of the ACTMus randomized control trial (Rose et al., 2018, 2022). Ethical approval was obtained from London-Camberwell St Giles Research Ethics Committee, UK (16/LO/0609).

### Intervention

To accommodate mobility issues, which could potentially reduce engagement with therapy, we configured the intervention for remote delivery as guided self-help. There is increasing evidence for the efficacy of remotely delivered interventions, including ACT, with a recent meta-analysis highlighting that self-guided online ACT (delivered through telephone or website) produced significantly greater outcomes than waitlist controls at post-treatment for variables including anxiety, depression, and quality of life for a range of mental health difficulties (Klimczak et al., 2023). The intervention was conceptualized for participants as offering them the opportunity to try out some “psychological skills” in everyday life to see if these prove helpful. A series of four skills was delineated to cover the breadth of psychological flexibility and presented to participants: mindfulness; unhooking (from thoughts); follow your values; and take an observer perspective. The intervention was delivered over 5 weeks and comprised four booklets, audio files containing mindfulness exercises, and five telephone or video calls delivered by a therapist trained in ACT (For more details on the composition of the intervention, see Rose et al., 2018, 2022).

### Recruitment

To be eligible for the ACTMus, study participants had to be adults (18 years and over) diagnosed with one of the following MDs that has been present for more than 6 months: LGMD, BMD, FSHD, or IBM. At the time of enrolment, they had to have reported moderate to severe symptoms of anxiety and/or depression on the Hospital Anxiety and Depression Scale (HADS; scoring ≥7 for either the anxiety or depression subscales). Participants were excluded based on the presence of health co-morbidities unrelated to MD, or treatments or situations that could make participation inappropriate (see Rose et al., 2018, 2022 for complete details).

For the nested qualitative study, all participants enrolled into the ACTMus trial were given the option of providing their consent to be contacted at the end of the study to discuss their experiences with a member of the research team. They consented to this being audio recorded so the research team could transcribe the conversation and explore common themes.

### Procedures and data collection

Upon completion of the intervention, all consenting participants randomized to the ACT plus standard medical care were contacted consecutively by a research assistant [RA (VE)]. This process and the qualitative analysis occurred before the trial data were analyzed (Rose et al., 2022). Enrolment continued until the point of data saturation, the point that no new themes were emerging.

The RA (VE) collected the qualitative data through semi-structured interviews consisting of a set of open questions and prompts (see Supplementary material) designed to elicit participants' perspectives on receiving ACT. The interview began with questions focused on participants' expectations pre-intervention and experiences during and after the intervention. All the interviews were conducted one-on-one by the same researcher over the telephone and recorded. Interviews lasted between 30 and 60 minutes.

Interviews took place between January and November 2017 and were recorded and transcribed verbatim. The RA who collected, transcribed, and analyzed the qualitative data was not involved in the development of the ACT intervention, nor its delivery, but was involved in the recruitment and data collection of trial participants. The RA had a master's degree and attended additional University-run training in NVivo. Further training and supervision were provided by the other authors with experience in the field.

### Data analysis

The RA transcribed interviews verbatim. Transcripts were analyzed using inductive thematic analysis (Braun and Clarke, 2006, 2021) using the software NVivo version 11 (http://www.qsrinternational.com/). Analysis began on completion of the first few interviews and proceeded iteratively. To obtain familiarity with the data, transcripts were read through several times by two researchers (VE and CV). These two researchers identified initial codes independently and met to resolve differences in coding. They then extracted codes and transformed them into themes in a recursive process. Once key themes were identified, they were discussed within the wider trial management group and refined.

It is important to acknowledge that although attempts were made to improve the reliability and validity of themes via independent coding, the pre-existing experiences of the researchers will naturally have shaped theme construction. The two analysts (VE and CV) identify as white, female, and able-bodied and work within the psychological field. The analysts discussed potential assumptions and biases to increase their own awareness of themes such as their beliefs around the value of psychological interventions, positions as individuals without chronic health conditions, and ethnocentric lenses. Both analysts used notes and reflective journaling to record ideas and enhance transparency in decision-making processes.

## Results

### Participants

A total of 27 consenting participants were contacted to take part. Five did not respond, one person stated they were too busy, and one person agreed, but a convenient time could not be arranged. As a result, a total of 20 (eight male, 12 female) participated. The mean age of participants was 52.1 years (SD = 15.15, range 29–77 years of age). Participants had one of four MDs (seven LGMD, nine FSHD, and four IBM). No participants had BMD. The mean duration of MD was 16.98 years (SD = 11.21, range 5–47 years). The baseline rating on the HADS for anxiety was a mean of 11.15/21 (SD =2.94, range 7–18), and for depression, a mean of 8.4/21 (SD = 3.12, range 2–15). Higher scores on the HADS demonstrate greater anxiety or depression, with a score of eight and above providing good sensitivity and specificity for indicating clinical significance (Bjelland et al., 2002). Further demographic information is displayed in Table 1.

**Table 1:** Demographic information.

	**Total (*n* = 20)**	**%**
**Gender**		
Female	12	60
Male	8	40
**Age (years)**		
20–29	2	10
30–39	3	15
40–49	3	15
50–59	4	20
60- 69	5	25
70+	3	15
**Ethnicity**		
British white	16	80
Other white	2	10
Asian	2	10
**MD diagnosis**		
LGMD	7	35
BMD	0	0
FSHD	9	45
IBM	4	20
**MD duration (years)**		
< 10	5	25
10–20	9	45
>20	6	30
**Level of function (ANA)** ^*^		
≤ 5	3	15
6−15	5	25
16–25	6	30
26–34	3	15
Missing data	3	15
**Anxiety and depression**		
HADS baseline anxiety score ≤ 8	18	90
HADS baseline depression score ≤ 8	14	70

^*^Adult Ambulatory Neuromuscular Assessment (ANA) is an adult version of the North Star Ambulatory Assessment that measures motor function (the higher the score, the better the patient can function).

### Findings from analysis

**Figure 1:**
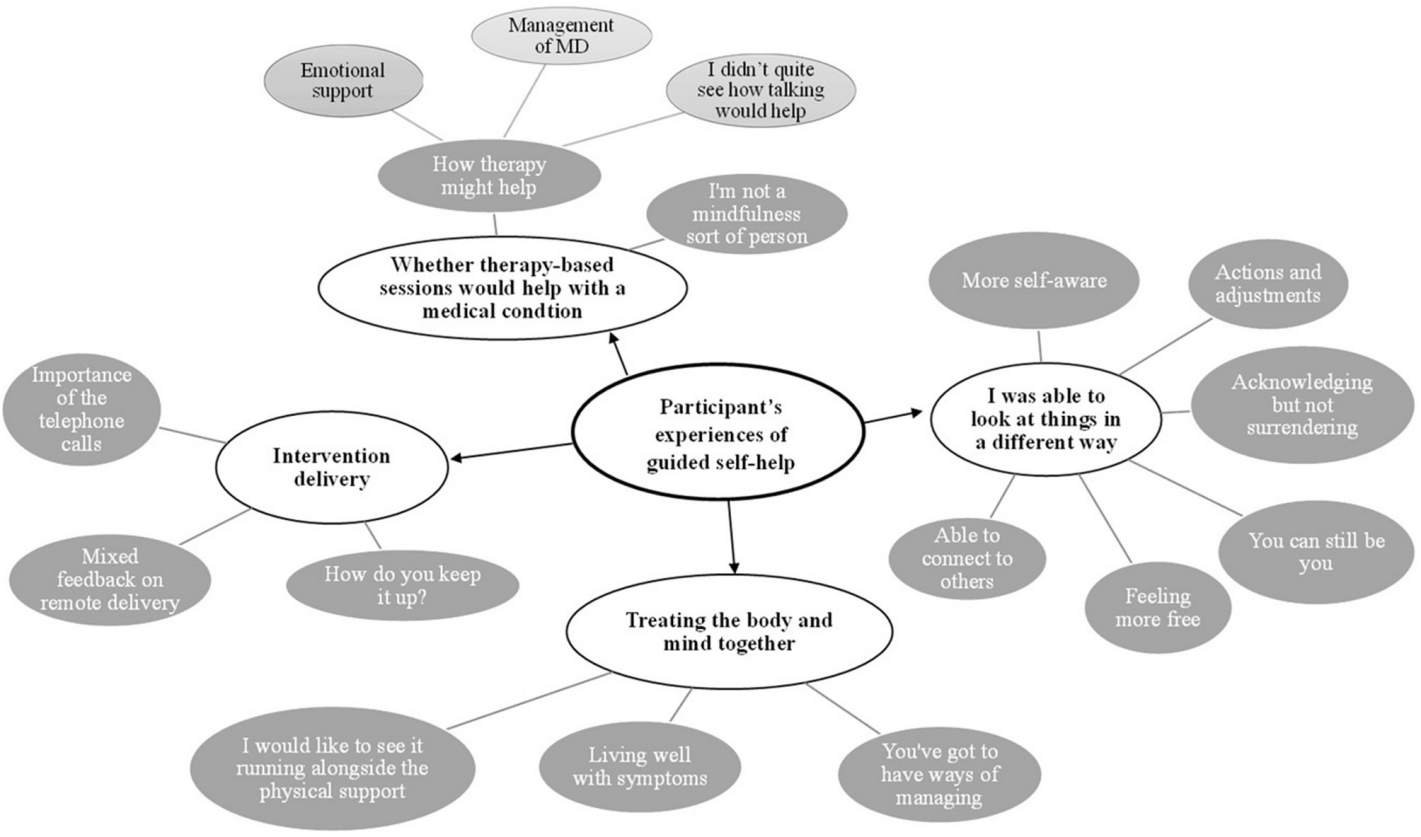
Thematic map of results.

Four over-arching themes were identified. These themes, alongside sub-themes, are mapped in Figure 1 and discussed in the text in more detail.

#### Theme 1: views on whether therapy sessions would help with a medical condition

The first theme describes participants' recollections of their thoughts and expectations before receiving the intervention. Most participants appeared to be unsure about what a psychological intervention could offer to people with MD, as one participant observed:

“*I wasn't really knowing what to expect, or whether a therapy-based sessions would help with a medical condition” (P11)*.

As denoted in this quote, it appeared that most participants were unclear about what therapy may involve and how it might help (rather than being skeptical). Indeed, participants largely reported having an “*open mind*” (P13): “*I was just very curious really. I just didn't know what to expect”* (P10).

##### How therapy might help

As discussed, at the outset of the intervention, the majority of participants reported being unsure about what the intervention would involve despite being open-minded. However, there appeared to be different ideas about *how* it could be of help.

(a) **Emotional support**

The first subtheme refers to participants who showed awareness of the emotional challenges that can come with MD. For them, the intervention provided hope as a new option for managing distress.

For example, one participant stated “*It just seemed interesting to me… because, sometimes I do struggle with the thought of the future and everything like that”* (P4).

Another participant felt they had reached a point where “*I needed something to help me or something that I thought could help me” (P9)*. This quote suggests a sense of hopefulness—that psychological support would be helpful. This was an undertone to several participant accounts. For example, “*I got quite excited about it, hoping it was going to be some sort of um quite um in depth therapy…which I thought I knew I needed at the time”* (P14).

As such, these participants were optimistic about the prospect of receiving a psychological intervention:

“*We definitely need some sort of psychological help- it's not that easy to be positive, it's not that easy to carry on with life because there are loads of times when you are upset, when it seems to be taking over your life”* (P1).

Therefore, this subtheme describes participants who had insight into the psychological challenges presented by MD and expressed hope that the psychological intervention would be helpful.

(b) **Management of MD**

The majority of participants perceived the intervention as another method for managing their MD.

“*I've got a condition that needs managing. And part of that management is how I think about it all”* (P8).

This is commensurate with medical narratives about managing a chronic condition. However, there was intrigue regarding how management could include the mind as well as the body: “*It was interesting to explore something more cognitive I suppose, as a form of management for a muscle wasting condition”* (P2).

The perceived aim of management was often described in practical and proactive terms, with one participant describing how they thought the intervention would involve “*…coming up with some ideas on how to deal with various kinda situations and that different sort of coping strategies and managing strategies” (P12)*.

Therefore, this group of participants perceived that the intervention aimed to offer “tools” to help them navigate life with MD, which is in slight contrast to participants who expected in-depth therapy to manage emotional challenges.

(c) **I didn't quite see how talking would help**

The final subsection of participants shared that they did not know how talking therapy could help.

“*I really didn't know what to expect, I had an open mind and think anything that's going to help is worth going for. I didn't quite see how talking would help”* (P13).

A couple of participants described doubt about the potential utility of the intervention. For example, one participant stated they were “*probably skeptical when I first started off… I was more of a physical person than a mental-type person for helping things.”*

This quote illustrates an uncertainty regarding how talking therapy could benefit individuals with a physical condition.

##### I'm not a mindfulness sort of person

Before starting the intervention, the mindfulness component of the intervention could elicit strong, sometimes negative, reactions.

Participants who had previous experience with mindfulness had strong responses regarding personal applicability: “I *was already aware that mindfulness is something that is not for me”* (P7) and “*I'm not a mindfulness sort of person… I did try it once before but it only lasted one session. Because I thought ‘God I can't be doing this”'* (P8).

Other participants did not have personal experience but viewed mindfulness negatively as a fad: “*…its become very much the flavor of the month… everyone talks about mindfulness don't they its everywhere”* (P8). As such, mindfulness was met with cynicism: “*you hear about this mindfulness… you think trying all that's a load of rubbish so the hardest part was actually buying into it. And having a go at it”* (P3).

Interestingly, a participant who by contrast did have experience with mindfulness also reported having to overcome preconceptions to engage in the intervention: “*I sort of had a certain arrogance to it like oh I've already done this, I know all about this kind of thing. And it took me a little bit of time to get over that arrogance and go well actually well no you still need to do it and um focus on it”* (P14).

Mindfulness therefore appeared to be a term that often elicited strong preconceptions and assumptions. Furthermore, many participants who had tried mindfulness held strong views about whether it was “for them” or not.

#### Theme 2: I was able to look at things in a different way

The following theme outlines changes participants reported after receiving the intervention.

(a) **More self-aware**

Participants reported that the intervention had helped them to approach their thoughts and feelings in new ways.

This involved becoming more aware of values, thoughts, and feelings. For example, “*I am certainly more self-aware. I have thought quite a lot about my values in life”* (P19) and “*I think prior to the course I was finding myself drifting off into other thoughts you know instead of working on one thought at a time”* (P15).

Participants then described how they responded differently to thoughts and feelings using techniques from the intervention. In relation to the “unhooking” skill, one participant described how “*It's like a dog with a bone and you worry about it and you keep going on and on and on and she taught me to and the module taught me to sort of um let it go to one side and when it keeps coming into your mind”* (P13).

Another participant described becoming able to notice and “step back” from difficult thoughts and feelings when required: “*Sometimes I can catch myself becoming angry and frustrated and upset about the sort of pain and the tiredness and the fatigue, I can just stop, sit down, and take a step back from it all where before I might just stay angry”* (P14).

Participants reported greater awareness of their own experiences (e.g., thoughts, feelings, and actions) and also their overarching goals and values. This awareness enabled them to actively and consciously employ skills to “step back” and take an “observer perspective” when problematic thoughts, feelings, or urges were present.

(b) **Actions and adjustments**

There were numerous reports of participants re-initiating activities that they had previously stopped due to the progression of MD symptoms.

One participant described how “*I've started going out more, I've started going to the gym, I've started going to meeting people although we'd stop for years”* (P3).

“*I was quite happy not to attend certain occasions because I would struggle to get home or what's happening if I fell over or do they have stairs and all this stuff. And then I managed to get myself to go several places I really enjoyed and really had a positive impact on my life”* (P1).

Participants reported that they were able to participate more fully in enriching activities by either making practical adjustments to the activity itself or changing their approach to it, to focus more on the process of the activity than the outcome.

“*I do try and pace myself more which means you still get tired but you don't get exhausted so I think it helped on that sort of pacing myself better”* (P10).

“*I think it's rather [than] setting goals and not getting them it's now more moving towards them. If you don't get there but you move towards it all the while”* (P3).

(c) **Acknowledging but not surrendering**

Participants reported a shift following ACT toward acceptance of physical changes, which led to enabled participation in activities they enjoy:

“*You've just got to learn to accept it, you've got to adapt and do things differently and that's one I suppose of the new challenges”* (P15).

Acceptance was not a passive resignation but an active process of facing difficulties (and potentially difficult thoughts and feelings) to undertake enriching activities: “*I think it's the acceptance that possibly there are some things that you won't be able to do so don't make yourself feel bad about them but you've got to look and say ‘well can I do it and if I can, can I do it in another way which makes it better and easier for me?”'* (P6).

This was often described as a pivotal change that participants experienced with ACT; as one participant put it, “*I think the most important part is acknowledging but not surrendering” (P8)*.

One participant had not disclosed their diagnosis to many of their friends due to fears about over-burdening them but found that opening up to these difficulties allowed them to reconnect with friends and engage in social activities:

“*You can't just pretend that's everything's fine can you, when it's not. And but you haven't got to sort of go miserable about it either—its worked out really well—I didn't want to burden anyone or feel guilty or anything and it hasn't worked out like that its worked out absolutely super… if I go see my friends I find it very difficult to open doors and they do it without thinking now because I've explained it to them so that was, as I say, that was invaluable to me”* (P10).

This participant described how the intervention helped them to open up to the present situation [“*I sort of tried to pretend it wasn't happening before but of course it is so I suppose that sort of quite an important lesson isn't it*” (P10)]. Furthermore, in getting closer to these difficult thoughts and feelings, they were able to take action and share their diagnosis, which ultimately allowed them to continue with social activities (“*I didn't want to stop going out with my friends so I told her and when she gets the coffees in she'll get me a straw I know that sounds a silly thing but the cups are usually too heavy*”). Thus, several participants reported that acknowledging their MD allowed them to feel less stuck and engage more fully in meaningful activities.

(d) **You can still be you**

This subtheme describes how many participants felt that they had regained a sense of their identity during the intervention. Receiving a diagnosis of MD, with accompanying changes in appearance, activities, and roles within the family or at work, often leads people to experience changes or fractures in their sense of self. However, through the intervention, they were able to reconnect with their sense of identity:

“*When you have your condition you kind of like think the old person died and kinda having to make do with the new one… but you know what I've actually always been there, you know, I've been able to be myself and that is really really good”* (P16).

For some, there were indications that MD led to reduced confidence or self-worth. However, as participants acknowledged difficulties and made adjustments, this enabled a renewed sense of self-esteem and self-worth. One participant describes the process as: “*acknowledging that you changed or that change in you but also acknowledging you still have value, but you have to sort of readjust your life to sort of enable yourself to do that and be the person you can be, because that's still within you”* (P8).

Another participant who stood for most of the day at work described not wanting to disclose difficulties with standing. They shared that they “*always thought I wouldn't be able to do the job if I didn't stand and stuff like that so I was torturing my legs, for no reason”* (P4). It seems that the intervention allowed them to think more flexibly and take an “*authoritative view at work”* (P4). They described how their workstation was modified, which “*let me accept the fact that I can still do my job and I can still be me and I can still do all the stuff I normally do but I just need to adapt a little bit”* (P4).

As such, for some participants, acknowledging difficulties and making adaptions allowed them to reconnect to lost aspects of themselves and regain a sense of identity and self-worth.

(e) **Feeling more free**

Many participants reported feeling freer or more motivated to undertake activities following ACT:

“*It kick-started me into being able to sort of to free myself really and… not hold myself back, you know because I can't walk anywhere”* (P8).

This sense of motivation or freedom was often described using physical terms: “*It sort of has made me a bit stronger”* (P6). Interestingly, these terms were used in conjunction with psychological skills: “*I found it very helpful and intriguing just the way that, you can use your mind to move forwards”* (P11).

There was also a sense that freedom could be obtained while accepting the use of mobility aids: “*going through this sort of enabled me to understand that although the cost to me [of using mobility scooter] was denial or my own sort of like thoughts of embarrassment or ‘it isn't me, I need to explain why I'm on it' and all this sort of malarkey that was a negative but the benefits is its opened my life up”* (P8).

In addition to the physical freedom due to increased mobility, some participants described a “*release”* from thoughts and feeling “*lightened” (P9)*. This appeared to be facilitated by defusion techniques taught in the intervention that showed participants methods to stop struggling with difficult thoughts: “*I found that this unhooking thing has really stayed with me and it's changing my life. Basically freeing up my time, free up my mind, free up my feelings—it frees up everything”* (P1).

Therefore, engaging with the intervention appeared to generate a feeling of liberation for several participants.

(f) **Able to connect to others**

Another by-product of engaging with the intervention was an increased connection with others. Whilst some participants reported no changes in the quality of their relationships following the intervention, some reported increased quantity and quality of interactions with loved ones:

“*There was one weekend I managed to spend enough record quality time with my family that my son said ‘oh what happened mummy you look much more happy than other times”'* (P1).

“*We've had some various tense moments between myself and my wife because it gets both of us after a while because I'm slowing changing my wife from being a full-time wife to a part-time carer and maybe eventually to a full-time carer so obviously there are difficulties for her… but I think things are getting easier, obviously if I'm not depressed then the bad vibes don't spill over”* (P15).

Alongside improved mood, participants described how feeling more “*relaxed*” (P15) and more themselves, “*I was able to laugh and joke and be you know exactly how I was*” (P16), increased their ability to connect with others.

#### Theme 3: treating the body and mind together

Our third theme relates to participants' accounts of what they learned post-intervention regarding the importance of being seen holistically as a person living with MD within healthcare treatments.

(a) **You have got to have ways of managing**

This subtheme describes participants' accounts of the importance of recognizing the psychological aspect of MD and the need for support in managing a long-term condition.

“*I was floating around lost and not really knowing what to do or having any tools to kind of help me get over this idea of coming to terms of this long-term chronic wasting disease and it was kind of swamping me a little bit and getting me quite down” (P14)*.

For some who, before receiving the intervention, had not felt a personal need for support, there was evidence of a change in opinion following the intervention: “*Personally I thought I was in an okay place. I didn't really see what benefits it might bring but having done it, I can see that maybe I wasn't quite in that okay place and it has brought more benefits. So I think it's been, it's been very good and I think it would help people.”* (P6).

They noticed the benefit of having coping skills through receiving a “*structure and a bag of tools”* (P14). These tools were viewed as helpful for navigating the physical challenges presented by MD:

“*You can't physically change us, you can't physically make life easier or better for us in that sense but psychologically you can kind of help us manage ourselves a bit better and if you can give us some tools that are useful for that then that's absolutely brilliant”* (P12).

(b) **Living well with symptoms**

The majority of participants reported that they were able to respond more effectively to symptoms and therefore experience a better quality of life alongside them, “*The symptoms haven't changed particularly. It's more in my mind, my mind-set has changed.”* (P9).

Again, there appeared to be an acceptance of symptoms and a commitment to acting in ways that would enhance activity and minimize suffering: “*I'm always gonna be in a certain amount of pain during the day …what I can do now is, is manage the way that I react to those pains… rather than pushing myself I'll take a step back and just deal with it. Rather than pushing myself and hurting myself even more.”* (P11).

One participant did provide an exception in reporting a change in their symptoms, “*Obviously your arms and legs are not going to work how they used to, but because you're more active, your muscles are not as, how can I explain, tight or they're I'd say they're not as bad”* (P16).

Through increased activity, this participant appeared to notice positive physical by-products of the intervention. However, for most participants, symptoms remained and what changed was how they were responding to, and living beyond, symptoms.

(c) **I would like to see it running alongside the physical support**

This subtheme describes accounts relating to the need for psychological support as part of holistic healthcare. Participants frequently emphasized that treatment should be holistic and focus on “*the body and the mind together as a human being”* (P1). For example, “*sometimes when you've got a physical condition the doctors just take that, and treat that, and forget that you know, a bigger element of living with it is in your head”* (P18).

It was widely reported that psychological support should be “*running alongside”* physical support, as one participant described:

“*We get physio and all sorts of other things which are specifically aimed at your physical ability—nothing else is targeted at mental ability, psychological ability to deal with it”* (P19).

One participant described how the intervention was a refreshing approach as healthcare appointments usually involved measuring physical decline as opposed to considering participants' values and plans for the future:

“*you go to your occupational health assessments and stuff like that and your, your constantly asked about how you are and what you're doing and all this stuff and your kinda treated a bit like cattle. So no one actually or nothing really comes along and says ‘let's get you moving forward again'. So it was a really refreshing approach to actually be embracing the idea of looking forward to the future. Because it's something that for a while I'd actually tried either to block out or not think about”* (P4).

#### Theme 4: intervention delivery

The final theme concerns participants' thoughts on the specifics of the intervention and its delivery.

(a) **Importance of the telephone calls**

The telephone calls were seen as a helpful, sometimes essential component of the intervention:

“*It wouldn't've worked unless you've got somebody on the other end of the phone”* (P13).

Regarding why the telephone sessions felt so important, one participant stated: “*I was just able to put it into real life scenarios by discussing it. The books were great and they make you think then when you want it in the real world it's nice to have that conversation with someone so you can apply the knowledge that you've just learnt”* (P11).

Although one participant said they would have preferred the intervention without the telephone calls as they found it too much “pressure” (P2), others described telephone sessions as helpful for engagement and motivation: “*It was a really useful benchmark each week to know that I was going to do the things but then there was going to be some sort of follow-up on them”* (P14).

Overall, the telephone calls were found to be helpful for applying techniques in the intervention to real-life scenarios, feeling understood, and providing a benchmark for completing and reviewing goals.

(b) **Mixed feedback on remote delivery**

There were mixed experiences regarding the remote delivery of the intervention. Some felt it would have been better face-to-face: “*…there's just something about being able to see somebody's face”* (P14).

Others felt that remote delivery was helpful due to convenience: “…*it's basically because of the hassle… especially if you work, to take a day off or a half day off you know it it's losing the point this was great it sort of fit around my life”* (P1).

There were also reports that remote delivery helped participants to speak more openly with the therapist: “*There's a certain level of anonymity over the phone and I think that for me personally that helped me to be more open and honest”* (P19). Similarly, another participant said that “*by not being able to see the person perhaps you're able to open up a little bit more.”* (P13).

Therefore, participants reported both positive (convenience and a helpful sense of anonymity) and negative (a sense of reduced connection with the therapist) consequences of remote delivery.

(c) **How do you keep it up?**

This subtheme refers to concerns regarding the brevity of the intervention. For some, the intervention seemed to end abruptly: “*How do you keep it up after receiving this last session with your psychologist? And I think it's a question as well for the other patients, whether we have enough strength to do it or not to forget it”* (P1).

Several described how further sessions or ongoing “check in's” may be required for the continued application of skills:

“*Obviously it's therapy so it can't go on forever you have to deal with it in a way by yourself but you know I think every now and again have a to have like a kind of a recap or refresh your mind and have someone to talk to is helpful”* (P7).

It was felt that continued support from a therapist may be particularly helpful following setbacks:

“*I think you will need to talk to me again if I have a nice big fall or something but I haven't had it for a while thank God but that can usually be very upsetting to think and then how can I move on from that”* (P1).

These quotes demonstrate participants' concerns about whether they will be able to maintain effective practice of the skills following completion of the intervention, given the brevity of the intervention and lack of ongoing contact with a therapist.

## Discussion

This nested qualitative study provided additional information on the experiences, impacts, and acceptability of the guided-self-help ACT intervention (Lewin et al., 2009; O'Cathain et al., 2014). The randomized controlled trial of the intervention led to moderate-to-large improvements in our primary outcome of QoL, and also secondary outcomes, including mood (Rose et al., 2022). The themes derived from the present study characterize how these improvements were experienced in everyday life of participants. Following ACT, participants described making several conscious changes. This included increasing their levels of activity to involve more activities they found personally meaningful, such as going to the gym or social events. Such activity appeared to be facilitated by other conscious changes, such as becoming more aware of their thoughts and feelings and finding ways to helpfully approach these experiences. For example, participants described a greater willingness to accept the emotions and thoughts that come with symptom progression, which enabled effective engagement with mobility aids. They described adapting their approaches to undertaking activities they find enriching. This involved prioritizing, pacing, or focusing on the process of living life consistent with their own values as opposed to simply focusing on reaching goals.

In addition to these conscious changes in behavior, participants mentioned some unexpected consequences following ACT. They described renewed ways of disentangling their identity from that of MD and beneficial impacts on key close relationships. Participants reported that everyday life included a quality of feeling more free, suggesting the experience of an increase in the breadth of choices available to participants. One participant reported experiencing improvements in muscle function and pain, alongside the sense that improvements in muscle function occurred via increases in physical activity. Indeed, in a randomized controlled trial of cognitive behavior therapy for fatigue in MD, perhaps by a similar mechanism, those receiving CBT showed improved muscle composition following therapy, as assessed with MRI (Janssen et al., 2016). Nonetheless, commensurate with the goals of the ACT intervention here, the majority did not report improvements in physical symptoms but rather expressed greater awareness that they could live well alongside physical symptoms.

Psychological flexibility is the change process specifically targeted by ACT (Hayes et al., 2006). There was some suggestion that participants changed their behavior in ways commensurate with psychological flexibility. For example, some participants described a general process of doing more activities they found fulfilling (engagement), with acceptance of concomitant challenging experiences (openness) and greater awareness of their thoughts, feelings, and choices (awareness). However, this slightly contrasts with changes in questionnaire measures of psychological flexibility observed in the randomized controlled trial where changes were of a small magnitude (Rose et al., 2022). This discrepancy could be due to limitations of quantitative measures of psychological flexibility, which may have accessibility/comprehensibility issues (Castle, 2019). It is also possible that the sub-sample of participants included in this qualitative study were those who experienced larger changes in psychological flexibility during the intervention.

This study provides further insight into the acceptability of the intervention and clues for enhancing uptake and engagement when implemented into clinical practice. Acceptability appeared to be high among our participants. People with MD live with a condition that in many instances comes with a high symptom burden and few available treatments. Yet, they reported being able to make meaningful changes—usually increases to their everyday activity—via this psychological intervention. Many of these participants suggested that psychological interventions should be included in their overall care, such as to help with the impact of physical symptoms. One might contend that such findings are to be expected given participants in the qualitative study also volunteered to be part of a trial of a psychological intervention. However, this finding is consistent with a previous questionnaire study of treatment and research needs in MD, where participants with MD highlighted a need for support for living well with the condition via psychological and associated interventions (Nierse et al., 2013). Since completing this study, evidence from a systematic review and meta-analysis suggests that online ACT produced significantly greater outcomes than waitlist controls at post-treatment for anxiety, depression, quality of life, psychological flexibility, and all assessed outcomes (i.e., omnibus effect), which were generally maintained at follow-up (Klimczak et al., 2023).

To enable access for those with mobility issues, the intervention was configured as remotely delivered guided self-help. Participants described generally positive experiences of remote delivery and therapist contact. Consistent with qualitative evaluations of remotely guided self-help ACT treatments with other health conditions (Köhle et al., 2017; Bendelin et al., 2020; Contreras et al., 2022), participants stated that contact with a therapist was necessary. They valued interactions that provided space for considering alternative perspectives on their experiences. In addition, although some would have preferred to receive therapy in person, the remote delivery was seen by many as beneficial. This was for practical reasons, such as fitting sessions into a busy day or keeping on track with therapy aims and goals. A surprising benefit of this remote delivery is that some found the apparent anonymity helpful—stating that it meant they could be more open about their experiences. Given the increasing use of remotely delivered interventions, there is surprisingly limited research into the role of anonymity and variables contributing to ways it might facilitate or hinder the therapeutic relationship.

There was mixed feedback as to the brevity of the intervention and participants' ability to maintain engagement. The intervention was conducted over 5 weeks, comprising self-help booklets and corresponding brief telephone calls with a therapist (Rose et al., 2018). Several participants reported concern about maintaining the progress they had made during the intervention and said they would have liked additional calls, asking whether “check-ins” could be integrated into their annual healthcare review. Our quantitative outcome data from the trial demonstrate a short-term impact of the intervention, with QoL and mood significantly better at 9 weeks. Our planned analysis of outcomes at 6 months will help understand whether benefits are maintained in the absence of additional “check-ins.”

Participants' pre-intervention expectations for receiving the ACT intervention provide clues to improve uptake. The emphasis on mindfulness as a part of the intervention appeared to elicit strong reactions. Several participants communicated skeptical attitudes toward mindfulness—seeing mindfulness as a fad—while several others felt certain from previous experience that mindfulness would not be helpful for them personally. Mindfulness practice, such as centring exercises, is a frequently used ACT method, largely as a way of promoting the present-moment-awareness aspect of psychological flexibility. However, other methods that do not require formal mindfulness practice can be used to promote present-moment awareness. For example, participants can be encouraged to consciously slowdown in emotive or important circumstances—to notice their options and patterns of thoughts, feelings, and action—or therapists can facilitate conversations that involve attending to ongoing experiences in granular detail (sometimes called “tracking” methods) (Villatte et al., 2015; Hayes, 2019).

## Limitations of the study

As a nested qualitative study, participants volunteered to participate in the ACTMus trial. Thus, the present group may have been more attracted to psychological intervention than the general population of people living with MD. Interviewing the wider population of people living with MD on the utility of psychological interventions could provide greater insight into ways to improve engagement with psychological interventions in MD. We only interviewed participants who completed ACT, which means we cannot generalize our findings to those who did not fully engage with the intervention. Although the response rate in this study was high (74%), it would be interesting to explore the experiences of those who decided to stop participating in ACT—this would perhaps give clues for improving engagement. Given that all discussions took place shortly after the completion of the intervention, future qualitative research would benefit from examining the longer-term engagement with skills engendered in the ACT intervention.

## Conclusion

Individuals with MD who participated in a guided self-help ACT intervention described making conscious changes to increase life-enriching activity following ACT, which came along with greater awareness of thoughts and feelings and new ways of engaging with experiences. Other potential benefits included the disentangling of participants' identity from MD, a better quality of relationship with others, and a general sense of feeling freer. There was a mainly positive evaluation of the remotely guided self-help configuration. While a small number would have appreciated in-person sessions, many saw practical benefits to the remote nature of the intervention and found the sense of anonymity helpful for talking more openly. The intervention was brief, and several reported that follow-up sessions may have been helpful. Many held negative preconceptions about the utility of mindfulness methods. For future implementation, it is worth considering the use of follow-up sessions and de-emphasizing the centrality of typical mindfulness exercises in the intervention. We cannot say whether these perspectives translate to other treatment modalities, such as CBT.

## Data availability statement

The datasets presented in this article are not readily available because of the potentially identifiable nature of the interviews. Requests to access the datasets should be directed to Trudie Chalder (Trudie.chalder@kcl.ac.uk).

## Ethics statement

The study was approved by the London-Camberwell St Giles Research Ethics Committee, UK (16/LO/0609). The study was conducted in accordance with the local legislation and institutional requirements. The participants provided their written informed consent to participate in this study.

## Author contributions

All authors listed have made a substantial, direct, and intellectual contribution to the work and approved it for publication.
