# Supplementary material for: Participant experiences of guided self-help Acceptance and Commitment Therapy for improving quality of life in muscle disease: a nested qualitative study within the ACTMus randomized controlled trial
Source: Front Psychol. 2023 Dec 1;14:1233526. doi: 10.3389/fpsyg.2023.1233526 (PMC10722278; doi:10.3389/fpsyg.2023.1233526)
Supplement: Supplementary file 1 [file Table_1.DOCX]

ACTMuS: Interview Topic Guide

Discussion points

1. Firstly can you tell me a little bit about your expectations before participating in the trial?

*Prompt 1: I’m interested in why you agreed to take part in the trial*

*Prompt 2: what you were initially expecting the treatment to involve?*

*Prompt 3: did you have any previous experience of ACT / mindfulness e.g. audio files?*

1. Next can you tell me about your experiences of having the ACT treatment

*Prompt 1: what kinds of things did the treatment involve?*

*Prompt 2: what were some of the good parts about receiving ACT?*

*Prompt 3: where there any challenges aspects to receiving ACT?*

1. Can you tell me what it was like to receive the intervention remotely?

*Prompt 1: Was it easy to access the booklets*

*Prompt 2: Was it easy to access / hear the audio files?*

*Prompt 3: Would you have made any changes to how the intervention was delivered?*

1. How did you find the materials themselves?

*4.A. How did you find the booklets?*

*Prompt 1.a: What was most helpful about having them?*

*Prompt 1.b: what was challenging about them?*

*Further prompts:*

- *How did you find the exercises / homework tasks?*
- *What was your experience of the metaphors– were they helpful?*
- *How easy to follow were the modules?*
- *How appropriate did you feel the style & language was?*
- *How was the length of the booklets and the timings for receiving them?*

*4.b. How did you find the audio files?*

*Prompt 1.a: Did they feel like a useful addition to the treatment?*

*Prompt 1.b: Did you find them helpful or not?*

*Further prompts:*

- *Did you like listening to them? How often did you listen to them?*
- *Other than just being relaxing do you think they are helpful?*
- *How did you find the content, were they easy to grasp?*
- *How was the sound quality?*

1. How/ what was your experience of the therapist?
   *Prompt 1: Were the telephone calls a useful addition to the materials?*

*Prompt 2: In what ways were they helpful / or not?*

*Prompt 3: Do you have any suggestions on how they could be improved?*

*Further prompts:*

- *How was the length of each session?*
- *Were there the right amount sessions?*
- *Do you think the sessions would have been different if in person… if yes, in what ways?*

1. Do you feel that anything has changed since receiving the intervention?

*Prompt 1: Do you feel like how you relate to your thoughts and feelings has changed? How?*

*Prompt 2: Has it changed the things you do?*

*Prompt 3: Have you noticed any other changes since the treatment e.g. your goals, work, relationships or even your symptoms?*

1. Have you been able to maintain any parts of the intervention?

*Prompt 1: Have you used the materials (or ideas from it) since finishing the intervention?*

*Prompt 2: Is there anything you have been able to maintain long-term?*

*If yes, what have you been doing? If no, what barriers were there?*

*Prompt 3: Did you find module 4 helpful in suggesting how to maintain the intervention? How could this be improved?*

1. Overall, looking back now on the treatment you received, what do you think about the use of ACT as a treatment for MD?

*Prompt 1: Do you think that the intervention provided helpful ideas or opened up ways to deal with the challenges relating to MD? What was particularly helpful?*

*Prompt 2: Were there any parts that you felt were not helpful/suitable for people with MD?*

*Prompt 3: Is there any way it could be better tailored to people with MD?*
